# Severe Primary Open-Angle Glaucoma and Agricultural Profession: A Retrospective Cohort Study

**DOI:** 10.3390/ijerph19020926

**Published:** 2022-01-14

**Authors:** Mathilde Grosselin, Leila Bouazzi, Thomas Ferreira de Moura, Carl Arndt, Maxime Thorigny, Stéphane Sanchez, Alexandre Denoyer

**Affiliations:** 1 Department of Ophthalmology, University Hospital, University of Reims Champagne-Ardenne, 51100 Reims, France; mathilde.grosselin@etudiant.univ-reims.fr (M.G.); tom_ferreira@hotmail.fr (T.F.d.M.); carndt@chu-reims.fr (C.A.); 2 Department of Biostatistics and Health Economics, University of Reims Champagne-Ardenne, CURRS, 51100 Reims, France; leila.bouazzi@univ-reims.fr (L.B.); maxime.thorigny@univ-reims.fr (M.T.); 3 Department of Medical Information, Hospital of Troyes, 10420 Troyes, France; stephane.sanchez@hcs-sante.fr; 4 Research Team EA4683 CARDIOVIR, University of Reims, 51100 Reims, France

**Keywords:** glaucoma, farmer, environment

## Abstract

While exposure to pesticides is a known risk factor for neurodegenerative brain diseases, little is known about the influence of environment on glaucoma neuropathy. We aimed to determine whether farmers are at higher risk of developing severe primary open-angle glaucoma (POAG). This retrospective cohort study (tertiary referral center, Reims University Hospital, France) included patients diagnosed with POAG in the last two years. Univariate analysis and adjusted multivariate logistic regression were performed to evaluate the association between agricultural profession and all recorded data. Glaucoma severity (primary outcome) and the number of patients who underwent filtering surgery (secondary outcome) were analyzed. In total, 2065 records were screened, and 772 patients were included (66 in the farmer group and 706 in the nonfarmer group). The risk of severe glaucoma was higher in the farmer group (adjusted odds ratio (aOR) 1.87, *p* = 0.03). More patients underwent filtering surgery in the farmer group in univariate analysis (*p* = 0.02) but with no statistical significance after adjustment (*p* = 0.08). These results suggest pesticide exposure may be a factor accelerating the neurodegeneration in POAG, although a direct link between the agricultural profession and the disease requires further extended studies to be demonstrated.

## 1. Introduction

Glaucoma is the leading cause of irreversible blindness in the world. Nearly 75% of glaucoma cases are primary open-angle glaucoma (POAG) [1]. POAG is a multifactorial pathology with some well-identified risk factors, such as age, intraocular pressure (IOP), first-degree family history with POAG [2], ethnicity, myopia [3], obstructive sleep apnea syndrome (OSAS) [4], and corneal thickness [5]. In contrast, other factors are still debated, such as sex, socioeconomic status, cultural level, diabetes, dysthyroidism, tobacco [6,7], alcohol, vascular factors, nutritional factors [8,9], neurotoxic factors [10], etc. In parallel, known risk factors for glaucoma progression are age, IOP, OSAS, and vascular factors (diabetes, systolic arterial hypotension [11]).

POAG is a chronic optic neuropathy characterized by retinal neurodegeneration [10]. Its pathophysiogenesis is complex and multifactorial [12]. The main therapeutic strategy is currently focused on treating ocular hypertension. Some studies have shown pesticides to be risk factors for neurodegenerative brain diseases, such as Alzheimer’s or Parkinson’s disease [13,14,15]. However, the association between neurotoxic agent exposure and the onset or worsening of glaucomatous neuroretinal degeneration has been little explored. Only one European case–control study, published in 2013, found a significant association between POAG and self-reported professional use of pesticides [16].

In the present study, the main objective was to investigate an association between the farming profession and POAG severity. Our primary hypothesis was that patients within the agricultural profession were more exposed to neurotoxic agents and would therefore be affected with more severe POAG cases than nonexposed patients. Secondary endpoints were other data reflecting the severity of glaucoma, including the morphological and functional damage of the optic nerve, the number of patients having undergone filtering glaucoma surgery, and the pharmacological antiglaucoma treatment.

## 2. Materials and Methods

### 2.1. Study Design

Patients over 40 years old diagnosed in our record for POAG at Reims University Hospital Center and who performed at least one automated visual field (VF) examination [17] (Humphrey with Swedish interactive threshold algorithm standard 24.2 testing) in the last two years were included. Exclusion criteria were any kind of glaucoma except POAG, peripheral iridotomy, nonglaucomatous optic neuropathy, thyroid-related orbitopathy [18,19], and any palpebral or retinal pathology altering the VF or pathologic myopia. We excluded high myopic patients only if they had myopic macular complications (myopic maculopathy, posterior staphyloma, choroidal neovascularization, chorioretinal atrophy) [20]. We also excluded patients with nonglaucomatous pathology leading to best-corrected visual acuity (BCVA) worse than 20/40, an unrealizable or uninterpretable VF, and patients unregistered under the social security system. Included patients were divided into two groups according to their profession: the farmer group and the nonfarmer group as a control group. A blinded assessor conducted data collection of the profession.

### 2.2. Judgment Criteria

**Figure 1:**
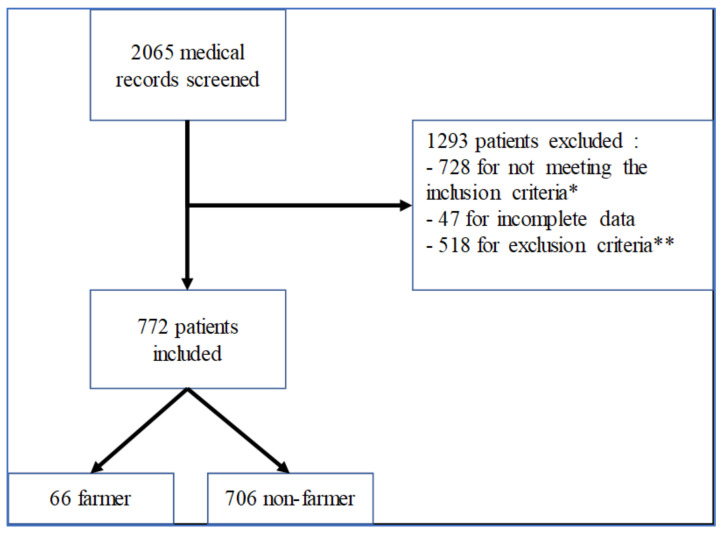
Flow chart of the study: * identifies patients followed for papillary excavation without ocular hypertension nor visual field impairment, patients followed due to a POAG family history with normal clinical examination, nonglaucomatous optic neuropathy, monitoring of treatment with drug-induced optic neuropathy, and nonorganic disorders; ** identifies any types of glaucoma except POAG, thyroid-associated orbitopathy, peripheral retinal damage or maculopathy, pathologic high myopia, and low BCVA unrelated to POAG.

The primary endpoint was POAG severity according to the profession (farmer group vs. nonfarmer group). Secondary endpoints were the number of patients receiving glaucoma filtering surgery, VF index (VFI), number of local treatments, and mean retinal nerve fiber layer (RNFL) thickness, according to the group. The specific terminology used in ophthalmology for this paper is detailed in Figure 1.

### 2.3. Data Collection

We collected data from medical records belonging to patients who had undergone examinations using a Humphrey visual field analyzer between September 2017 and December 2020 [21]. We used the glaucoma staging system (GSS) proposed by Mills et al. (a modified version of the Hodapp–Anderson–Parrish System) to determine POAG severity [22,23]. This classification is based on mean deviation (MD), number and location of points depressed at *p* < 0.01 and *p* < 0.05 on the pattern deviation plot, visual acuity, and the glaucoma hemifield test. Severe POAG was defined as a score > 3 according to the GSS classification. The Humphrey visual field test was interpretable when meeting the following parameters: fixation loss rate <20%, false-negative rate <15%, and false-positive rate <15%. In order not to bias the conclusions, only one eye was included per patient, the most affected according to the GSS classification. We chose the eye with the lowest MD index if both eyes were at the same severity stage. For ocular hypertension (stage 0), we included the eye with the highest IOP. The spherical equivalent of the included eye was extracted in patients who did not receive cataract surgery and were without senile cataract leading to index myopia. For each patient, we retained the most-extended follow-up period (at least, the follow-up duration at Reims University Hospital Center and at best, the disease duration if it was noted in the medical record). We collected POAG family history, but this variable could not be analyzed due to missing data.

### 2.4. Ethical Considerations

The study design (from retrospective observation) was based on a medical database that did not require patient consent, according to French legislation [24]. This study was performed in compliance with national legislation regarding epidemiological studies (Declaration N°2206749 v 0). Moreover, in accordance with national ethical directives, the requirement for written informed consent was waived because the study was strictly observational and all data were blinded [25]. According to the French Public Health Code, this research also did not require an ethical committee, which was confirmed by the Committee for the Protection of Persons Est I (Dijon, France). Patients were informed that the study was being carried out via the hospital’s registry of ongoing studies.

### 2.5. Statistical Analysis

Continuous variables were expressed as means ± standard deviation in normal distribution; otherwise, we used medians and interquartile ranges (IQR). The normality of distributions was assessed graphically and by using the Shapiro–Wilk test. Categorical variables were expressed as percentages. Continuous variables were compared according to the profession using Student’s *t*-test (or the Mann–Whitney U test in cases of deviation from normal distribution). Categorical variables were compared using the χ^2^ test (or Fisher’s exact test when the expected cell frequency was <5). The association between agricultural profession and glaucoma severity was evaluated using a univariate logistic regression. Age, sex, and factors that were significantly associated with MSA (*p*-value <0.10) were included in a multivariate logistic regression. Odds ratio (OR) were derived from the model as effect sizes. Missing data were considered missing completely at random and were replaced by the mean or the median (according to the distribution) [26]. Statistical testing was performed at the two-tailed α level of 0.05. Data were analyzed using the SAS software package, release 9.4 (SAS Institute, Cary, NC, USA).

## 3. Results

### 3.1. Patient Population

We analyzed 2065 medical records of patients initially diagnosed for glaucoma and who underwent one automated visual field at least in the last two years. Of those, 728 patients did not meet the inclusion criteria, 47 patients had incomplete medical records (that required data or had a loss of visual field report, for example), and 518 patients presented with at least one exclusion criteria. Finally, we included 772 patients in our study, 66 belonging to the farmer group and 706 to the nonfarmer group, as shown in Figure 1.

### 3.2. Main Endpoint

Patients’ characteristics are detailed in Table 1. There was no statistically significant difference between the two groups regarding any epidemiological data. In contrast, the number of severe POAG cases was significantly higher in the farmer group (28.8% versus 17%; OR 1.97; 95% confidence interval (CI), 1.12–3.48; *p* = 0.02). After adjusting for age, sex, and intraocular pressure, the difference remained statistically significant (adjusted OR (aOR) 1.87; 95% CI, 1.05–3.34; *p* = 0.03), as shown in Table 2.

**Figure 2:**
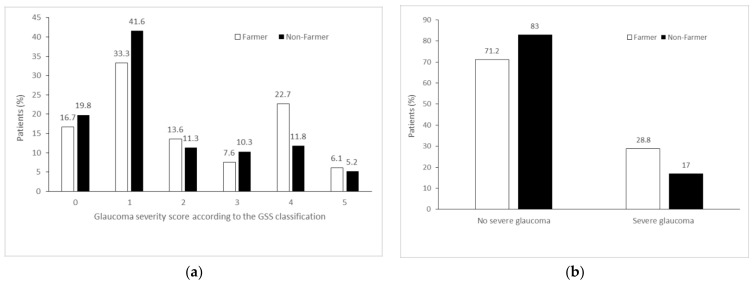
Distribution of the glaucoma severity score is displayed (**a**) according to the proportion of severe glaucoma and (**b**) according to farmer and nonfarmer group. GSS = glaucoma staging system. Severe glaucoma is defined for a GSS score >3.

The histograms of patient distribution for each group, according to the stage of POAG severity, showed an asymmetric distribution of data, as shown in Figure 2. In both groups, the highest peak was in stage 1, which means that in each group, most patients had mild-severity POAG. In the farmer group, there is a two-peak distribution curve corresponding to stages 1 and 4 of the disease.

### 3.3. Secondary Endpoints

More patients underwent filtering glaucoma surgery in the farmer group (11 patients or 16.7%) than in the nonfarmer group (55 patients or 7.8%). This difference was statistically significant in univariate analysis (OR 2.37; 95% CI, 1.17–4.78; *p* = 0.02), but multivariate analysis, with adjustments, resulted in a loss of significance (aOR 2.28; 95% CI, 1.12–4.64; *p* = 0.08). No statistically significant difference between groups was observed in mean RNFL thickness, number of treatments, or in VFI, as shown in Table 2.

## 4. Discussion

The main aim of our study was to investigate an association between the agricultural profession and severe POAG. While previous studies established a link between the agricultural profession and neurodegenerative diseases, particularly Alzheimer’s and Parkinson’s diseases, very little data exist on POAG. A European case–control study (Photograf study) on 339 POAG cases (exposure) and 339 ocular hypertension (controls) matched according to age-conducted questionnaires based on self-reported memory to evaluate the occupational use of pesticides and other chemicals. After adjusting for age, sex, and illness duration, a significant association between POAG and use of pesticides during working life (OR 2.65; 95% CI, 1.04–6.78; *p* = 0.04) was found. Likewise, more patients with POAG were employed in the agricultural profession in their lifetime (OR 2.57; *p* = 0.009) and used pesticides during their occupation (9.0% versus 4.2%; OR 2.54; *p* = 0.02) [16].

POAG is a neurodegenerative disease for which the pathophysiology is, to date, only partially understood [27]. Many authors see in POAG a retinal analogy with the neurological pathologies of Alzheimer’s and Parkinson’s diseases and raised the hypothesis of common pathophysiological mechanisms [28,29]. In Parkinson’s disease, the responsibility for direct and indirect toxicities of α-synuclein aggregates on dopaminergic neurons in substantia nigra is now well established [30]. At the retinal level, Parkinson’s disease can reduce the ganglion-cell-layer thickness and increase the outer-plexiform-layer thickness due to a possible local accumulation of α-synuclein aggregates [31].

Genetic and environmental risk factors for Parkinson’s disease have been identified [13]. Various epidemiological studies have shown that occupational exposure to pesticides is a risk factor for POAG [16], Alzheimer’s [14], and Parkinson’s diseases [13,15]. Since 2012, Parkinson’s disease can, under certain conditions, be recognized as an occupational disease in French farmers [32]. The main classes of pesticides implicated in the genesis of Parkinson’s disease are pyridine derivatives (paraquat), dithiocarbamates (maneb), inhibitors of the mitochondrial respiratory chain (rotenone), and organochlorine compounds (dieldrin) [33]. Several pathophysiological models attempted to explain their links with Parkinson’s disease: oxygenated free radicals production facilitating the aggregation of α-synuclein, which is directly toxic for dopaminergic neurons; inhibition of mitochondrial membrane complexes I and III; and involvement of cytochromes c in the formation of α-synuclein fibrils [33,34]. In addition, there might also be microglial activation, either primary by direct pesticide toxicity [33] or secondary [35] by neuronal damages, which would then lead to dopaminergic neuronal apoptosis of the mesencephalic substantia nigra.

Surguchov et al. [36] described synuclein presence within the human retina with a predominance of γ-synuclein in the ganglion cell layer and retinal nerve fibers [37]. Both α- and β-synuclein are found mainly in the inner plexiform layer and therefore would have a synaptic location [36]. To date, the links between γ-synuclein and neurodegenerative diseases have been poorly studied. However, a significant drop in the level of γ-synuclein appears to be sufficient to induce apoptosis of retinal ganglion cells, testifying its essential role in the homeostasis of these cells [38]. Similarly, Surgucheva et al. found a collapsed intraretinal γ-synuclein level in glaucomatous optic neuropathy [39].

Based on the results of our study and the data from the literature regarding a link between pesticides and neurodegenerative diseases, we suggest the possibility of a pathophysiological association between pesticide exposure and glaucomatous neuropathy. Due to the induction of intraretinal neuronal damage, local neuroinflammation by microglial activation would exacerbate the apoptosis process of retinal ganglion cells and therefore cause glaucomatous neurodegeneration. One strength of our study was data collection conducted by a blinded assessor of profession, allowing for objective analysis of judgment criteria and prevention of assessment and ranking biases. Collecting the profession (objective data) rather than self-reported exposure to pesticides allowed us to avoid possible memory bias. Finally, we raise a plausible pathophysiological hypothesis, and our results are consistent with other studies on this subject.

The main limitation of our study is that a farmer profession may not indicate the level of exposure to pesticides, which depends mainly on crop type and farmer duties. A more precise evaluation of the dose–response for each patient would have strengthened our results. Conversely, patients belonging to the nonfarmer group may have been exposed to neurotoxic substances depending on their specific professions (e.g., pesticide control worker and landscape worker) or outside their professional settings when living in the vicinity of areas treated with pesticides [40,41,42]. However, the French State has imposed minimum distances to be respected between residential areas and pesticide-application areas [43]. Indeed, it would also have been interesting to (i) detail the putative at-risk professions in the nonfarmer group and (ii) analyze occupational exposure more precisely, for example, by using the MATPHYTO crop/exposition matrices [44], which are databases that assess, retrospectively and exhaustively, pesticide exposure for a type of agricultural crop in a given geographical area. These matrices could help us target agricultural subpopulations that are most at risk, such as winemakers. We have shown a statistical link between two factors, but it is difficult in such an epidemiological study to establish a causal link between substance exposure and disease onset. Other hypotheses could partially explain our results and, therefore, could lead to residual confusion. With long working hours, farmers probably have fewer regular medical checkups and lower medication adherence than the general population. For the same reasons, POAG cases could be diagnosed at later stages in these patients. Farmers may also be more isolated, as social deprivation has been associated with more severe POAG cases at the time of diagnosis [45]. However, in France, patients have free access to the public health care system, and a glaucoma screening is systematically offered to them from the age of 40.

## 5. Conclusions

This work originally reports that being a farmer is a risk factor for severe POAG. Our study is the first to primarily focus on the impact of agricultural profession on the severity of the neuropathy in POAG, but prospective studies are required to assess the temporality between occupational exposure and POAG onset or its aggravation. An observational study including the entire French population and the level and kind of pesticide exposure is currently being conducted in our department to try to answer these crucial points.

## Figures and Tables

**Table 1:** Characteristics of patients in farmer and non-farmer groups.

	Overall *N* = 772 Subjects	Farmer *N* = 66	Nonfarmer *N* = 706	*p*-Value
**Age (years): means ± SD**	69.7 ± 10.8	71.7 ± 11.2	69.6 ± 10.8	0.12
**Gender**				0.32
Male	388 (50.3)	37 (56.1)	351 (49.7)	
Female	384 (49.7)	29 (43.9)	355 (50.3)	
**Intraocular pressure (mmHg): median (IQR)**	16 (14–19)	16 (15–20)	16 (14–19)	0.05
**Selected (most affected) eye**				0.44
Right	351 (45.5)	27 (40.9)	324 (45.9)	
Left	421 (54.5)	39 (59.1)	382 (54.1)	
**Corneal thickness (μm): means ± SD**	533.1 ± 42.2	536.8 ± 34.6	532.7 ± 42.9	0.38
**Spherical equivalent (diopters): median (IQR)**	0.25 (0.00–0.75)	0.25 (0.00–0.75)	0.25 (0.00–0.75)	0.72
**OSAS**	78 (10.1)	6 (9.1)	72 (10.02)	0.77
**Diabetes**	159 (20.6)	11 (16.9)	148 (21.0)	0.44
**Duration of follow-up (months): median (IQR)**	43 (23–66)	49 (25–69)	42 (23–66)	0.33

Data are stated as percentages, unless otherwise indicated. IQR = interquartile range; OSAS = obstructive sleep apnea syndrome; SD = standard deviation.

**Table 2:** Primary and secondary endpoints according to the agricultural profession.

	Farmers *N* = 66	Nonfarmers *N* = 706	OR (95% CI)	*p*-Value	Adjusted OR † (95% CI)	*p*-Value
**Primary endpoint**						
Severe glaucoma ^1^	19 (28.8)	120 (17.0)	1.97 (1.12–3.48)	0.02	1.87 (1.05–3.34)	0.03
**Secondary endpoints**						
Surgery	11 (16.7)	55 (7.8)	2.37 (1.17–4.78)	0.02	2.28 (1.12–4.64)	0.08
RNFL thickness (μm): means ± SD	71.2 ± 20.7	73.7 ± 20.6	0.99 (0.98–1.01)	0.34	1.00 (0.98–1.01)	0.52
Treatment: median (IQR)	2 (1–3)	2 (1–3)	1.16 (0.93–1.44)	0.20	1.14 (0.91–1.43)	0.26
VFI (%): means ± SD	73.1 ± 32.4	79.0 ± 28.9	0.99 (0.99–1.00)	0.18	0.99 (0.99–1.00)	0.24

Data are stated as percentages, unless otherwise indicated. ^1^ Severity was defined by a score >3. † Adjusted for age, sex, and intraocular pressure. CI = confidence interval; OR = odds ratio; RNFL = retinal nerve fiber layer; VFI = visual field index.

## Data Availability

All data is available upon request to the corresponding author.
